# The link between inflammation, bugs, the intestine and the brain in alcohol dependence

**DOI:** 10.1038/tp.2017.15

**Published:** 2017-02-28

**Authors:** S Leclercq, P de Timary, N M Delzenne, P Stärkel

**Affiliations:** 1 Institute of Neuroscience, Université Catholique de Louvain, Brussels, Belgium; 2 Louvain Drug Research Institute, Metabolism and Nutrition Research Group, Université Catholique de Louvain, Brussels, Belgium; 3 Institute of Experimental and Clinical Research, Laboratory of Hepato-Gastroenterology, Université Catholique de Louvain, Brussels, Belgium; 4 Department of Hepato-Gastroenterology, Cliniques Universitaires Saint-Luc, Brussels, Belgium

## Abstract

In recent years, some new processes have been proposed to explain how alcohol may influence behavior, psychological symptoms and alcohol seeking in alcohol-dependent subjects. In addition to its important effect on brain and neurotransmitters equilibrium, alcohol abuse also affects peripheral organs including the gut. By yet incompletely understood mechanisms, chronic alcohol abuse increases intestinal permeability and alters the composition of the gut microbiota, allowing bacterial components from the gut lumen to reach the systemic circulation. These gut-derived bacterial products are recognized by immune cells circulating in the blood or residing in target organs, which consequently synthesize and release pro-inflammatory cytokines. Circulating cytokines are considered important mediators of the gut–brain communication, as they can reach the central nervous system and induce neuroinflammation that is associated with change in mood, cognition and drinking behavior. These observations support the possibility that targeting the gut microbiota, by the use of probiotics or prebiotics, could restore the gut barrier function, reduce systemic inflammation and may have beneficial effect in treating alcohol dependence and in reducing alcohol relapse.

## Introduction

Alcohol dependence has traditionally been considered a brain disorder in which the alteration of various neurotransmitters and their receptors in specific areas such as the brain reward circuit plays a major role in the development of the addiction.^1^ The neurotransmitter systems implicated include dopamine, serotonin, opioid peptides, glutamate and γ-aminobutyric acid (GABA), which are involved in positive and negative reinforcement processes that participate in the motivation for drug seeking and maintenance of alcohol use after the development of dependence.^2^ Pharmacological approaches that have been developed to treat alcohol use disorders mainly target these neurotransmitter systems (Table 1). These drugs, although somewhat improving the clinical outcomes and relapse rate, only display a small effect size,^3^ suggesting the possible involvement of other, more peripheral, biological processes.

Over the past few years, numerous studies have supported that inflammation might be important for the development of psychological disorders such as depression,^4, 5, 6^ anxiety,^6^ alcohol craving^7, 8, 9^ as well as cognitive dysfunction,^6^ which all characterize the pathopsychological facet of alcohol dependence. Interestingly, several studies have demonstrated that inflammation affects multiple neurotransmitter systems^10^ by, for instance, increasing the expression and function of serotonin transporter and GABA receptors in the hippocampus,^11^ or by inducing the enzyme indoleamine 2,3-dioxygenase, which breaks down tryptophan, the precursor of serotonin, into kynurenine and other downstream metabolites (kynurenic acid and quinolinic acid) that influence glutamatergic neurotransmission.^12^ On the other hand, immune cells are capable of synthesizing and releasing neurotransmitters such as GABA.^13^ GABA exposure has been shown to inhibit the inflammatory response *in vitro*^14, 15^ and *in vivo*, in a mouse model of obesity.^16^ This cross-talk between the neurotransmitters and the immune system can influence the response to anti-depressant drugs. Indeed, the anti-inflammatory effect may be one of the many mechanisms by which antidepressants exert their therapeutic effect.^17^ A meta-analysis has shown that systemic tumor necrosis factor (TNF)α levels decrease over time in treatment-responsive patients, but remained elevated in treatment non-responders.^4^

The origin of inflammation in alcohol dependence and other psychiatric diseases is however not yet clear. Several factors such as psychological stress, early life stress, obesity, diet (high-fat diet, imbalance of n-6/n-3 polyunsaturated fatty acids), oxidative stress linked to ethanol metabolism and alcohol-induced liver steatosis could be involved. Another potential source of inflammation is the gut microbiota, a huge community of microorganisms colonizing the intestine, that interacts with the host and influence many aspects of physiological processes such as body homeostasis, metabolism and immunity.^18, 19^ Recent evidence suggests the gut microbiota as a new important factor in health and disease, including neuropsychiatric disorders.^20^ Complex pathways, involving endocrine, immune and neural signaling, mediate the communication between the intestinal bacteria and the central nervous system (CNS), thereby influencing brain function, mood and behavior.^21^ Changes in the composition of the gut microbiota have been observed in various psychiatric disorders such as autism,^22, 23^ depression,^24^ Parkinson’s disease^25^ and alcohol dependence,^26, 27^ and interested readers may refer to excellent reviews describing the gut–brain axis and its potent role for mental illnesses.^21, 28, 29^

This review will focus on the role of gut microbiota—as an important regulator of the immune response—in the pathophysiology of alcohol addiction. New potential strategies targeting the gut, and not directly the brain, will be suggested to improve health and psychological symptoms of alcohol-dependent patients.

### Animal models vs human studies in alcohol research

Animal models have proved very useful in addressing mechanistic questions on the role of inflammation in alcohol dependence and studies on germ-free (GF) mice have been extremely instrumental in introducing the concept that the gut microbiota may largely influence behavior. However, data obtained in animals should be interpreted with caution especially with regard to the changes in human behavior and the possibility to extrapolate them to human disease should carefully be checked in well-designed clinical studies.^30, 31^ Overall, animals have a natural aversion for alcohol and do not develop a true addiction as observed in humans. Moreover, the rate of ethanol metabolism and elimination is five times greater in mice than in humans.^32^ Alcohol administration to rodents (for example, Lieber-DeCarli liquid diet, Tsukamoto–French model of continuous intragastric ethanol infusion via gastrostomy) requires artifices that do not directly mimic human drinking patterns, and the damages alcohol causes to the organs in animals represent only a part of the spectrum of that observed in humans (for example, induction of little or even no inflammation at all^33^). In addition, numerous differences exist in the characteristics of the murine and human immune system, with, for instance, the balance of circulating lymphocytes and neutrophils,^34^ the numbers of Toll-like receptors (TLRs) expressed at the immune cell surface (13 in rodents and 10 in humans)^35^ and the species-specific differences in TLR regulation following, for example, stimulation with lipopolysaccharide (LPS),^36^ a component of Gram-negative bacteria considered a key factor in the development of alcoholic liver disease in rodent models by stimulating the TLR4–CD14 pathway.^37^ However, to our knowledge, it remains unknown whether differences in gut microbiota composition between mice and humans are consistent with a differential pattern of TLR expression and stimulation. Although imperfect, animal models are still useful if their limitations and weaknesses are understood and taken into consideration. We will hence present, in this review, data obtained in both animal and human studies.

### General effects of chronic alcohol abuse on the innate and adaptive immune systems

Alcohol consumption is known to alter both innate and adaptive immune responses.^38^ However, the effects of alcohol on the immune response are largely influenced by the dose and the length of exposure (acute vs binge vs chronic). Although numerous studies have shown that acute alcohol exposure inhibits the pro-inflammatory response *in vitro* and *in vivo*,^39^ chronic alcohol abuse results in the activation of the immune response. Alcoholic patients show a general dysregulation of the immune system, which make them more susceptible to bacterial (pneumonia and tuberculosis) or viral (HIV and hepatitis C) infections.^40^ They exhibit elevated levels of circulating pro-inflammatory cytokines^8, 9, 41^ as well as signs of inflammation in various peripheral organs (gastrointestinal tract, lung and liver) and in the brain.^39^

**Figure 1:**
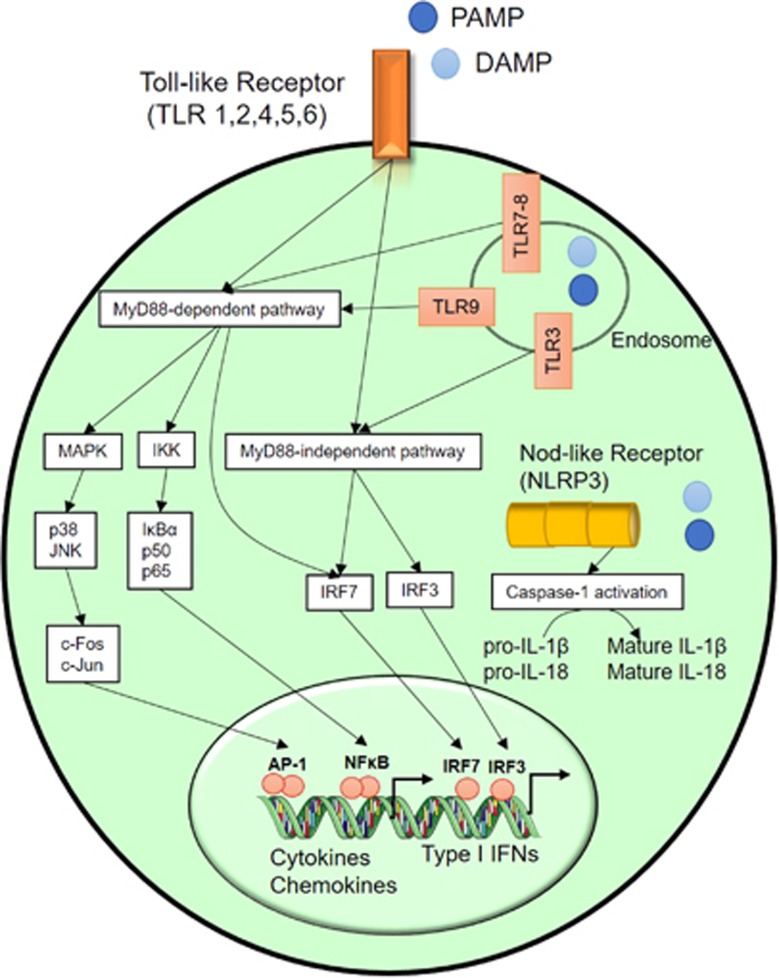
Activation of Toll-like receptors (TLRs) and Nod-like receptors (NLRs) by pathogen-associated molecular patterns (PAMPs) and damage-associated molecular pattern (DAMPs). Extracellular or intracellular binding of PAMP and DAMP to their receptors activates inflammatory pathways, dependent or independent of MyD88, which leads to the nuclear translocation and DNA binding of transcription factors (NFκB, AP-1 and IRF), resulting in the upregulation of pro-inflammatory cytokines, chemokines and type I interferons. Numerous bacterial, viral and host-derived ligands can activate the NLRP3 inflammasome complex constituted by the enzyme pro-caspase-1. Activation of caspase-1 is necessary to produce biologically active cytokines IL-1β and IL-18. IL, interleukin.

Innate immune cells, comprising monocytes/macrophages, dendritic cells, neutrophils, eosinophils, mast cells and the natural killer lymphocytes, express pattern recognition receptors, which include TLRs and Nod-like receptors.^42^ These receptors are located on the cell surface or within the cells and can sense, recognize and bind pathogen-associated molecular patterns that mainly consist of bacterial or viral products such as LPS from Gram-negative bacteria, peptidoglycans (PGN) and lipoteichoic acid from Gram-positive bacteria, flagellin, nucleic acids (DNA and RNA) or damage-associated molecular patterns (=danger signals resulting from damage to the body’s own cells). The interaction of pattern recognition receptors with their specific ligands activates intracellular signaling pathways resulting in the production of a plethora of cytokines, chemokines and type 1 interferon,^43, 44^ (Figure 1). Innate immune cells also express major histocompatibility complex molecules to present pathogen-derived molecules (that is, antigens) to naive T lymphocytes, thereby initiating the adaptive immune response. The latter can be subdivided into cell-mediated immunity (CD4^+^ T-helper cells, CD8^+^ T-cytotoxic cells and regulatory T cells) and humoral immunity (B cells and plasma cells that produce immunoglobulins).^45^

Numerous studies demonstrated systemic inflammatory changes in alcoholics.^41^ However, the results reported in the literature are highly heterogeneous and even contradictory. To date, there is no consensus on what inflammatory cytokines are actually changed by alcoholism itself. Discrepancy is likely due to coexisting comorbidities, including the stage of liver disease, the nutritional status, obesity, metabolic disorders, age, drug use, methodology used (*in vivo* vs *ex vivo*), the status of ethanol intake at the moment of the study (active drinkers vs sobers) all of which could influence the production of cytokines. For instance, monocytes from alcoholic hepatitis patients show a higher spontaneous NFκB activity associated with greater production of cytokines and chemokines compared to healthy controls,^46, 47, 48^ whereas another study showed spontaneous increased production of pro-inflammatory cytokines in monocytes of alcoholic patients without liver disease but not in alcoholics with liver disease.^49^ Peripheral blood T lymphocytes are slightly increased and express activation antigens (CD25, CD69 and HLA-DR), which correlate positively with alcohol intake in patients that do not suffer hepatitis.^50^ Despite a lower number of B lymphocytes, these patients usually have higher blood levels of circulating immunoglobulins (IgG, IgA and IgM) possibly reflecting an abnormal regulation of antibody production and/or a manifestation of auto-immunity or molecular mimicry.^40, 51, 52^ All these data demonstrate an altered immunity in alcoholics with a persistent activation of T cells that may result in an inappropriate immune response to pathogens and impaired host defense. The gastrointestinal tract is the primary site of interaction between the microorganisms and the immune system, and recent evidence supports that disturbances in the bacterial community result in dysregulation of the immune cells.^20^

### How can intestinal bacteria communicate with the brain to influence mood and behavior?

In addition to influence host physiology, metabolism and immunity, accumulating data indicate that the gut microbiota also communicates with CNS, and thereby influence brain function and behavior.^21^ In line with this assumption, GF mice exhibit multiple spontaneous brain changes including hyperactivity of the hypothalamus–pituitary–adrenal axis,^53^ leaky blood–brain barrier (BBB) permeability,^54^ axon hypermyelination^55^ as well as behavioral changes such as reduced anxiety,^56^ impaired social interactions^57^ and cognition.^58^ The mechanisms underlying the bidirectional communication between the gut and CNS are multiple and highly complex involving immune, neural and endocrine pathways. Below we summarize those for which experimental and human data suggest their potential role in alcohol addiction with a particular emphasis on immune pathways.

### The gut microbiota as a potential initiator of immune system activation

The gut, and more particularly the gut microbiota, is a major source of pro-inflammatory agents that have the capability to stimulate immune cells of target organs.

#### Breakdown of the gut barrier function in alcohol dependence

By using different probes and markers, several independent studies have shown an alteration of the gut barrier function, also referred to as ‘leaky gut’, in rodents exposed to alcohol^59, 60, 61, 62^ and in alcohol-dependent subjects.^9, 27, 63, 64, 65^ The intestinal barrier is mainly composed of enterocytes, tightly bound to their neighboring cells owing to apical junctional proteins (claudins, occludin and zonula occludens) known as tight junctions and adherens junctions.^66^ The barrier function is reinforced by a protective mucus layer elaborated by the goblet cells and antimicrobial substances, such as regenerating islet derived (Reg)3b and Reg3g secreted by the Paneth cells, which shape the composition of the intestinal microbiome.^67^ In addition, numerous immune cells in the lamina propria play an essential role in defending the intestinal mucosa against invading bacteria.^66^

How alcohol, and its primary metabolite acetaldehyde, causes gut leakiness is not yet well-established but multiple mechanisms have been proposed. They include myosin light-chain kinase activation,^68^ NFκB activation,^69^ upregulation of intestinal circadian clock gene expression,^70^ overexpression of miRNAs that inhibit tight junctions translation^71^ and reactive oxygen species production.^30^ In addition, pro-inflammatory cytokines such as TNFα have been demonstrated to downregulate tight junctions expression and cause disruption of the gut barrier.^72, 73^ High expression of TNFα in macrophages of the lamina propria has been found in duodenal biopsies of alcoholics.^74^ Those patients were also characterized by alteration of the thickness of the duodenal mucus layer^75^ as well as by decreased Reg3g protein expression.^76^

#### Intestinal dysbiosis in alcohol dependence

Accumulating evidence demonstrates that a new factor, the gut microbiota, is involved in ethanol-induced leaky gut. Animal studies have shown that improvement of ethanol-induced gut barrier dysfunction can produce beneficial effects on distant organs. Although data are scarce with regard to the gut–brain interaction, this has been clearly demonstrated for the gut–liver axis where both improvement of intestinal barrier integrity as well as liver injury can be achieved by using antibiotics,^59^ dietary fibers^60^ or probiotics^61^ that all modify the composition of the gut microbiota. This indicates that therapeutic strategies targeting the gut microbiome may be effective in the treatment of alcohol use disorders.

Culture-independent next-generation sequencing techniques^77^ make possible the identification of qualitative and quantitative microbial changes induced by chronic alcohol abuse. Gut dysbiosis (that is, alteration of the gut microbiota) has been reported in rodents chronically exposed to ethanol^74, 75, 76, 78, 79, 80^ as well as in the colonic mucosa and fecal samples of alcoholics^26, 27, 81^ (Table 2). Dysbiosis was characterized by numerous bacterial taxa changes such as decreased levels of the anti-inflammatory bacteria *Faecalibacterium prausnitzii* and *Bifidobacterium*,^27, 81^ and increased abundance of Proteobacteria,^26, 78^ a major group of Gram-negative bacteria. Increase in fecal pH induced by ethanol exposure has been proposed to drive the overgrowth of pathogens such as Proteobacteria.^78^ The latter represents an important source of LPS that can easily cross the hyper-permeable gut mucosa to reach the systemic circulation and activate TLRs of immune cells in blood and target organs resulting in the production of pro-inflammatory cytokines. Increased blood LPS levels in alcoholics have been shown in several studies^9, 65, 82^ without, however, establishing a clear correlation with the severity of the addiction or psychological and/or brain modifications. Long-term alcohol abuse is also associated with alteration of the gut microbiota functionality,^27, 83^ with drastic changes in specific metabolites (particularly phenol and indole) secreted by the bacteria that could also participate in gut barrier dysfunction.

#### Relationship between intestinal permeability and dysbiosis

**Figure 2:**
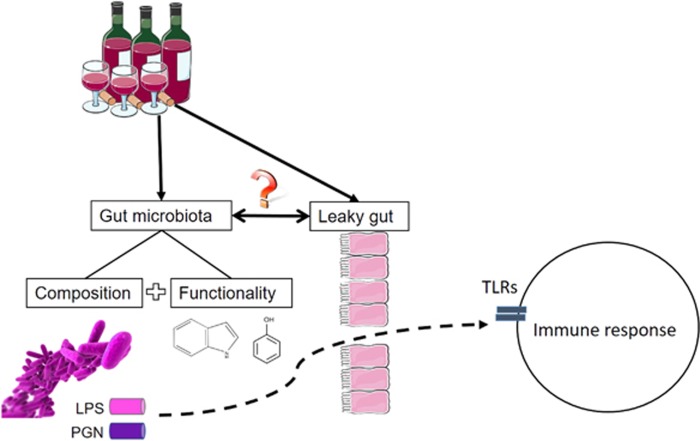
Chronic alcohol abuse is associated with gut barrier alteration, dysbiosis and immune activation. Alcohol-dependent subjects present with increased intestinal permeability (leaky gut) and altered gut microbiota composition and functionality. This favors the translocation of gut-derived bacterial components, such as lipopolysaccharides (LPS) and peptidoglycan (PGN), from the gut lumen to the systemic circulation and other organs. Bacterial ligands are recognized by Toll-like receptors (TLRs) expressed by immune cells and induce an inflammatory response.

Although alcoholics presented with increased intestinal permeability and gut dysbiosis, questions remain on a cause and effect relationship between these two outcomes (Figure 2). Indeed, it is not clear whether alcohol consumption first induces alteration of the gut microbiota composition, which leads to leaky gut (via for instance, a decreased abundance in bacteria—such as, for example, *Bifidobacterium*—that reinforce the intestinal barrier function), or whether alcohol induces gut barrier alteration, which in turn results in enteric dysbiosis (through, for instance, decreased expression of antimicrobial peptides such as Reg-3b and Reg3g (refs. 76, 79)).

Animal models of chronic alcohol exposure have shown that ethanol-induced leaky gut is a very early event that occurs within 2 weeks of alcohol feeding,^62^ whereas ethanol-induced gut dysbiosis occurs later, after 8 or 10 weeks.^78, 80^ Data in humans suggest a more complex interplay between microbes and gut permeability. Intriguingly, two independent studies have shown that only some, but not all, actively drinking alcoholic subjects, presented with alteration of the gut microbiota,^26, 27^ which correlated with increased intestinal permeability.^27^ Indeed, patients with dysbiosis had higher intestinal permeability, whereas patients without microbial alterations did not, despite heavy alcohol consumption.^27^ In addition, both studies have shown that sober alcoholics still exhibited gut dysbiosis,^26, 27^ despite a total restoration of intestinal permeability after >2 weeks of abstinence.^27, 63^ Therefore, it seems that (1) alcohol consumption alone is not sufficient to increase intestinal permeability or to induce gut dysbiosis and (2) dysbiosis without alcohol exposure (in sober alcoholics) can co-exist with a normal gut barrier function. These findings suggest that both alcohol consumption and gut dysbiosis are necessary to induce leaky gut. In line with this hypothesis, recent experiments of fecal transplantation from human alcohol-dependent subjects to GF mice fed with alcohol revealed that the gut microbiota might play a causative role in the modulation of intestinal permeability and in the development of alcoholic liver disease.^84^ Mice harboring the gut microbiota from a patient with severe alcoholic hepatitis developed greater intestinal permeability, higher bacterial translocation and more severe liver inflammation than mice harboring the gut microbiota from a patient without alcoholic hepatitis, despite the same amount of alcohol consumed. These observations support the hypothesis that the gut microbiota contains pro-inflammatory signals, which likely derive from pathobionts. Although this study brings crucial information in gut–liver axis research, further studies using fecal transplantation are urgently needed not only to confirm a causal relationship between dysbiosis and gut permeability in alcohol dependence but also to better define the role of the gut microbiota in the modulation of neurological processes that ultimately influence behavior of patients.

#### The rational for using pro- and prebiotics in alcohol dependence

Decrease in beneficial bacteria such as *Lactobacillus* and *Bifidobacterium* has been shown in animal exposed to alcohol and in alcohol-dependent subjects. Consequently, restoration of these bacteria could represent a potential target to improve alcohol-related diseases. In experimental models of alcoholic liver disease, modulation of the gut microbiota by the use of probiotic *Lactobacillus GG* or dietary fibers reduces gut leakiness, endotoxemia, inflammation and improves liver function.^60, 61, 85^ In humans, a 5-day supplementation with probiotics *Bifidobacterium bifidum* and *Lactobacillus plantarum 8PA3* during alcohol detoxification had greater effect on the reduction of liver enzymes than abstinence alone^81^ and a 4-week administration of *Lactobacillus casei Shirota* to alcoholic cirrhosis patients improved the neutrophil phagocytic capacity.^86^ Although these studies tempt to show a benefit in term of liver disease, no data are currently available sustaining a potential benefit of probiotics for brain alterations and psychological symptoms in alcoholics. However, in otherwise healthy subjects, previous interventional studies have demonstrated beneficial psychotropic effects of probiotics with improvement of anxiety and depression,^87^ cognitive reactivity to sad mood (due to reduced rumination and aggressive thoughts),^88^ stress-associated abdominal symptoms^89^ and brain activity.^90^ In major depressive disorder patients receiving probiotics, improvement of depression scores was associated with a reduction of inflammatory biomarker hsCRP.^91^ The mechanisms underlying the psychotropic effect of probiotics in humans has not yet been elucidated, whereas in rodents, a strain of *Lactobacillus* has been shown to decrease depression and anxiety-like behaviors through the activation of the vagus nerve.^92^

Prebiotics are selectively fermented ingredients that result in specific changes in the composition and/or activity of the gastrointestinal microbiota, thus conferring benefit(s) upon host health.^93^ They exert their health effects through the production of beneficial metabolites such as short-chain fatty acids (acetate, propionate and butyrate) with antimicrobial activity, lower intestinal pH to inhibit pathogen growth such as Proteobacteria, reinforce the colonic defense barrier and exhibit anti-inflammatory properties.^94^ Prebiotics might be safer and more efficient than probiotics since they have a broad effect on the gut microbial ecosystem and can change the abundance of >100 bacterial taxa.^95, 96, 97^ *Faecalibacterium prausnitzii* and *Bifidobacterium*, which are drastically decreased in alcoholics,^27, 81^ exhibit anti-inflammatory properties^98, 99^ and their abundance increased after consumption of prebiotics galacto-oligosaccharides or inulin-type fructans in healthy volunteers^100^ and in obese patients.^96^ In rats, consumption of prebiotics has been associated with neurochemical changes in the CNS with increased hippocampal brain derived neurotrophic factor and glutamate receptor expression,^101^ which are involved in the regulation of numerous behaviors including anxiety/depression, cognitive performance and addiction.^102^

Antibiotics are another way to modulate the gut microbiota but they have been associated mainly with negative outcomes. No improvement of endotoxemia or liver function has been shown in alcoholics receiving the broad-spectrum antibiotic paromomycin for 4 weeks.^103^ In rodents, depletion of the gut microbiota by antibiotics induced changes in brain neurochemistry and cognitive impairment,^104, 105^ and, importantly, modified the behavioral response to a psychostimulant drug.^106^ In this latter study, oral, but not intraperitoneal, administration of antibiotics resulted in increased sensitivity to the behavioral effects of cocaine, which could be reversed by the administration of short-chain fatty acids. Intraperitoneal injection of minocycline, known to alter neuroimmune and cytokines expression in the brain, was found to reduce ethanol intake but no link with the gut microbiota has been examined in this study.^107^ Finally, in humans, the use of antibiotics has been associated with an increased risk of depression and anxiety.^108^

Prospective, randomized, placebo-controlled, well-designed clinical trials are definitely needed to evaluate the effect of modulation of the gut microbiota (by pro- or/and prebiotics) on alcohol dependence and, more particularly, on the psychological symptoms (anxiety, depression and craving) as well as on the different behavioral aspects of alcohol addiction, such as impulsivity, compulsivity, alcohol-seeking behavior, stress, cognitive and executive functions.

### Systemic inflammation as a means of the gut to communicate with the brain in alcohol dependence

Alcohol-dependent subjects present with chronic low-grade systemic inflammation as witnessed by elevated plasma levels of TNFα, interleukin (IL)-1β, IL-6, IL-8, IL-10 and hsCRP even in the absence of actual bacterial or viral infection.^8, 9, 41^ *In vivo*, ethanol is likely not sufficient to induce the peripheral inflammatory response observed in alcoholics, as elevated plasma pro-inflammatory cytokines are still found after a period of sobriety,^9^ suggesting that stimuli other than ethanol might challenge the immune system. Furthermore, inflammation is thought to be involved in the development of other psychiatric disorders where alcohol does not play a role.^109, 110, 111^ The origin and mechanisms contributing to systemic inflammation in alcohol dependence as well as in other neuropsychiatric diseases are not yet fully understood but increasing evidence suggests that the gut microbiota might take part to this process.

#### Activation of PBMCs by gut-derived bacterial toxins contributes to systemic inflammation

Peripheral blood mononuclear cells (PBMCs) represent an essential defense barrier against gut-derived bacterial products entering the bloodstream and may therefore contribute to the chronic low-grade systemic inflammation in alcohol-dependent subjects. Mechanistic analyses performed in naturalistic conditions have revealed that LPS and to a higher extent PGN, derived from Gram-negative and Gram-positive bacteria, respectively, can contribute to the activation of PBMCs.^8^ Indeed, LPS receptors TLR4 and CD14 as well as PGN receptor TLR2 expression and activation were found to be higher in PBMCs of alcoholics compared to healthy subjects. In addition to elevated plasma PGN levels, the PBMCs expression of NOD2 (an intracellular receptor that binds the bioactive structure of PGN), was found to be increased in alcoholics. Activation of the transcription factor AP-1 and Nod-like receptor protein 3 inflammasome in PBMCs was suggested to contribute to the elevated plasma levels of IL-1β and IL-8. By contrast, downregulation of TNFα and IL-6 in PBMCs suggests that these two latter cytokines, which are actually increased in the plasma of alcoholics,^8, 9^ might originate from other sources, like different circulating immune cells (for example, neutrophils) or peripheral organs such as the liver or the gut wall itself that are also directly in contact with ethanol and bacterial components. Detailed analysis of the inflammatory pathways in these two organs and whether they release pro-inflammatory cytokines into the blood in response to alcohol abuse would further enhance our knowledge concerning the origin of systemic inflammation in alcoholics.

#### Systemic inflammation correlates with psychological symptoms of alcohol dependence

The induction of behavioral symptoms by peripheral inflammation is a pivotal element of the sickness behavior theory.^112^ In brief, this theory supports that peripheral infections lead to the activation of the innate immune system, through the recognition of bacterial or viral compounds by TLRs, and consequently the production of pro-inflammatory cytokines.^43^ The cytokines are able to reach the brain^113^ and subsequently induce the three components generally observed during sickness, that is, fever, a neuroendocrine response and behavioral changes such as fatigue, lassitude, inability to concentrate, irritability, loss of appetite and withdrawal from normal social activities. When inflammation persists, as occurs in chronic inflammatory diseases, sickness behavior may transform into depressive symptoms.^5, 114, 115^ A large body of experimental and clinical evidence supports a causal role for inflammation in the development of various psychiatric disorders. For instance, a substantial proportion of cancer or hepatitis C patients treated with IFN-α and IL-2 develop psychiatric symptoms including depression.^116, 117^ Patients suffering major depression, who present with high baseline levels of circulating pro-inflammatory markers, showed improvement of depressive symptoms when treated with infliximab, a monoclonal antibody that binds and blocks the actions of TNFα.^118^ Modulation of the immune system and induction of inflammation during gestation in rodents and primates has also been implicated in the development of autism- and schizophrenia-related behavior.^119, 120^ Finally, intravenous injection of LPS to healthy humans induced increased serum levels of TNFα, IL-6 and cortisol, which were associated with depressed mood, increased anxiety and decreased memory performance.^6, 121^

Alcohol use disorders frequently occur with other psychiatric conditions. Almost one-third of alcoholics present with mood disorders (for example, major depressive disorders and bipolar disorders) and 37% of the patients do have an anxiety disorder.^122^ Very often, affective disorders precede the onset of alcohol addiction. Importantly, even if a proportion of alcoholic patients are not diagnosed for mood or anxiety disorders, their levels of depressive and anxious symptoms are significantly higher than in healthy subjects and that is why anxious and depressive symptoms, craving, as well as cognitive dysfunction are considered important (neuro)psychological markers of addiction severity. Pro-inflammatory circulating cytokines were found to positively correlate with scores of depression, anxiety and alcohol craving^9^ in alcoholics. The association between inflammation and alcohol craving was confirmed in another study,^8^ where the improvement of craving scores during short-term alcohol withdrawal correlated with the decrease in IL-1β and IL-8 expression in PBMCs of patients—with IL-8 being considered the best predictor of craving.^9^ By contrast, the anti-inflammatory cytokine IL-10 was negatively correlated with psychological scores and craving at the end of a 3-week detoxification.^9^

The limitation of these correlational data in humans is that they do not address causality between changes in peripheral inflammation and modifications in craving or alcohol consumption. By contrast, data obtained in rodent models argue in favor of a cause–effect relationship showing, for instance, that genetic deletion of inflammatory genes is associated with a change in alcohol preference and consumption.^123^ Nevertheless, as negative emotional states and alcohol craving play a crucial role in negative reinforcement, a major factor favoring drug-seeking behavior and relapse,^124^ these observations highlight the possibility that reducing inflammation could help patients to improve their psychological well-being and subsequently reduce the probability of relapse. Modulation of the gut microbiota by the use of pro- or prebiotics is one potential way to reduce inflammation^125^ and there is now clinical evidence that probiotic supplementation reduces psychological symptoms, stress and changes brain connectivity in humans. So far, the effect of pro- or prebiotics on addictive behavior has not been investigated.

### The vagus nerve: a potential link between peripheral and central inflammation

**Figure 3:**
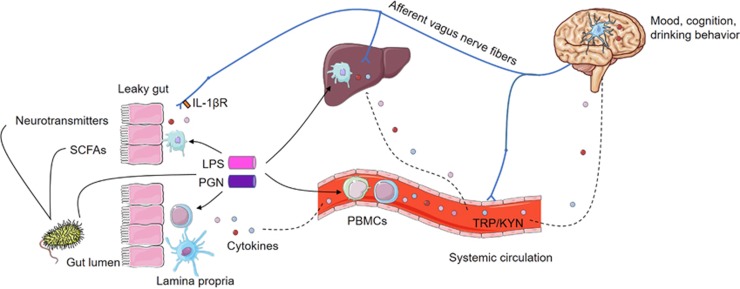
Gut-to-brain pathways of communication. Gut-derived bacterial components (LPS and PGN) activate the immune cells localized in the systemic circulation (peripheral blood mononuclear cells—PBMCs), or in target organs such as the gut or the liver that also release pro-inflammatory cytokines. These peripheral circulating cytokines are important mediators of the gut–brain axis as they can reach the central nervous system (CNS) and induce *de novo* the synthesis of cytokines within the brain. Brain cytokines are thought to mediate changes in mood, cognitive function and drinking behavior. Afferent vagus nerve fibers express the IL-1β receptor and can also convey the peripheral inflammatory message to the CNS and influence brain function and behavior. Other potential mechanisms of gut–brain communication involving the secretion of neurotransmitters, short-chain fatty acids (SCFAs) and the tryptophan/kynurenine (TRP/KYN) are also depicted. IL, interleukin; LPS, lipopolysaccharide; PGN, peptidoglycans.

The vagus nerve, which innerves the organs of the abdominal cavity, is also a well-established route of neural communication between the periphery and the CNS. Induction of brain cytokines expression and sickness behavior following peripheral administration of LPS has been shown to be mediated by the vagus nerve.^126, 127^ Afferent vagus nerve endings express receptors to IL-1 and prostaglandins,^128^ and consequently appear to be important for relaying information about the immune status to the brain. Pro-inflammatory cytokines produced or released in blood or peripheral organs (for example, gut and liver) activate vagal transmission and induce *de novo* synthesis of cytokines in projection regions of the vagus nerve, particularly the nucleus of the tractus solitarius^129^ (Figure 3). The vagus nerve is also an important way of communication between intestinal bacteria and the CNS.^130^ Subdiaphragmatic vagotomy has demonstrated that the reduction of anxiety- and depression-like behaviors observed in mice fed with beneficial bacteria *Lactobacillus rhamnosus* JB1 and *Bifidobacterium longum* are actually vagal-dependent.^92, 131^

The role of the vagus nerve in alcohol dependence has not been examined so far. Studies in animal models and in actively drinking and sober alcoholics should be conducted to determine the activation status of the vagus nerve, the possible relationship with systemic inflammation and its potential to influence brain function and behavior in alcoholism.

### Induction of innate immune genes in the brain participates in the neurobiology of addiction

Chronic alcohol abuse also activates brain immune cells that results in neuroinflammation and epigenetic changes, which could favor addictive behavior.^132, 133^ An important question remains whether ethanol, a lipophilic molecule that crosses the BBB, induces directly an immune response in the brain through its action on neurons, microglia and astrocytes, or whether peripheral blood cytokines reach the brain to stimulate immune cells of the CNS that in turn produce cytokines. The latter option involves several immune-to-brain communication pathways^113^ including the circumventricular organs that are devoid of a functional BBB, an active transport of cytokines through the BBB, the secretion of inflammatory mediators (PGE2) by perivascular macrophages and brain endothelial cells that express TLRs and IL-1 receptors. Microglia and astrocytes are part of the brain innate immune system, as they express pattern recognition receptors and can consequently respond to pathogen-associated molecular patterns and damage-associated molecular patterns by producing pro-inflammatory cytokines.^134^ Repeated exposure to alcohol leads to a long-term activation of microglia and astrocytes that secrete pro-inflammatory cytokines resulting in neuronal damage, cell death and behavioral changes such as anxiety-like behavior and impaired cognitive function.^132, 135, 136^ By using knockout mice and small interfering RNA, researchers found that activation of TLR4 in microglia and astrocytes following ethanol exposure is crucial to induce neuroinflammation^137, 138^ and BBB impairment.^139^ Moreover, peripheral injection of LPS induces long-lasting increase in ethanol drinking,^140^ suggesting a major role of TLR4 in alcoholic disease. By contrast, a recent comprehensive study across multiple laboratories, using different animal species and different models of drinking patterns, has shown that TLR4 was not a critical determinant of excessive drinking.^141^

The involvement of the brain immune system in the modulation of alcohol consumption and addictive behavior has also been shown by studies reporting upregulation of TNFα, IL-1β, IL-6 and MCP-1 expression in several brain areas of rodents chronically exposed to ethanol,^135, 138, 142, 143, 144, 145^ as well as by studies using genetic deletion of immune genes. Indeed, mutant mice lacking chemokine (Ccl2/MCP-1) or cytokine (IL-6) genes or their receptors displayed reduced ethanol preference and consumption.^123, 146^ By contrast, transgenic mice overexpressing IL-6 showed increased alcohol preference.^147^ In humans, increased expression of innate immune genes (MCP-1, TLR2, TLR3, TLR4 and high-mobility group box 1 (HMGB1), a danger signal exerting cytokine-like effects) has been shown in the brain of alcoholics collected post-mortem.^148, 149^

Ethanol exposure could also contribute to the neurobiology of addiction by altering the glutamate signaling through immune mechanisms.^132^ In brain slice cultures, TNFα has been shown to reduce glutamate transport,^150^ thereby increasing extracellular glutamate levels^151^ that lead to an hyperexcitability state which could inactivate the frontal cortex, with possible influences on mood and cognition.

### Additional mechanisms potentially involved in gut-to-brain communication

Additional mechanism, yet incompletely understood, might also be taken into consideration when exploring gut–brain communication (Figure 3). For instance, intestinal bacteria can synthesize neurotransmitters,^152^ such as GABA, serotonin and dopamine, which are important regulators of the brain reward circuit. Gut bacteria can also release short-chain fatty acids following the fermentation of dietary fibers. These compounds have neuroactive properties that could directly influence brain function and behavior.^153^ The tryptophan/kynurenine pathway is regulated by several enzymes tightly controlled by the immune system.^154^ Under inflammatory conditions, this pathway is activated and tryptophan, the precursor of serotonin, is converted into kynurenine, which in turn is converted into other neuroactive metabolites. Depletion of serotonin and production of kynurenine metabolites that could cross the BBB and exert neurotoxic actions is also one potential means of communication between the periphery and the brain. A study has suggested that the anti-depressant effect of probiotic *Bifidobacterium infantis* could be due to its modulation of the tryptophan/kynurenine pathway.^155^ Finally, some bacteria (for example, *Bacteroides* and *Clostridium perfringens*) display molecular homology with neuropeptides (for example, neuropeptide Y, α-MSH and ghrelin), which may result in the production of auto-antibodies that bind both the bacterial proteins and neuropeptides. Auto-antibodies display a dual function depending on their affinity for the peptide and can therefore serve as a peptide carrier or by contrast result in peptide neutralization.^52^ The potential role of molecular mimicry and auto-antibodies has been suggested in some psychiatric disorders (for example, eating disorders and major depression) and correlations have been found between levels of auto-antibodies against neuropeptides and anxiety scores^52, 156^

## Conclusion and perspectives for future research

Increasing evidence attributes a role for the gut microbiota and gut-derived microbial components as immune modulators that could contribute to the development of gut, liver, systemic and brain inflammation in alcohol dependence. Although this review particularly focuses on inflammation as a way for the gut to communicate with the brain, one has to keep in mind that other pathways involving, for example, the vagus nerve, neurotransmitters and metabolites definitely participate to the complex bidirectional gut–brain interactions.^21, 29^ Although tremendous amount of experimental and clinical work on ethanol-induced leaky gut, endotoxemia and gut dysbiosis has been performed in the gut–liver axis, very few studies have been dedicated to analyze the effect of gut microbiota on brain and behavior in alcohol dependence, although being a psychiatric disease.

Circulating blood cytokines have the capacity to convey the peripheral inflammatory message to the CNS eventually resulting in the alteration of mood and behavior including drinking behavior in alcoholics. Recent data obtained in a large cohort of alcohol-dependent patients implicated PBMCs in the release of IL-1β and IL-8 into the blood.^8^ However, other important cytokines such as TNFα and IL-6 likely originate from other type cells or other target organs such as the gut or the liver. Analysis of the inflammatory pathways in organs affected by alcohol abuse would help to clarify the potential sources of systemic inflammation in alcohol dependence and finally enhance our mechanistic comprehension of the disease.

Bacterial components originating from the intestinal microbiota, concurrently with alcohol exposure, seem to be strong inducers of the immune response. In addition to its modulatory effect of the immune system, the gut microbiota has also the ability to influence brain function and behavior. However, to date, studies almost exclusively base their conclusions on correlations between gut modifications, inflammation and behavioral changes. A formal proof of a direct cause and effect relationships is still lacking in humans. Two recent elegant studies^84, 157^ using human stool sample, from alcoholics or from major depressive disorder patients, transferred to GF mice found that specific dysbiosis contributes to the development of alcoholic liver disease or of depressive-like behavior, also emphasizing the feasibility of using fecal transplantation from human to mouse. As members of the gut microbiota exerting protective function such as *F. praustnizii* and some strains of *Bifidobacterium* are found exclusively in human gut microbiota, the use of humanized mice (GF mice receiving human microbiota) might be a more appropriate model to investigate the involvement of the gut microbiota in health and disease, despite several limitations linked to this procedure.^158^ One is tempted to speculate that the use of humanized mice could better mimic human behavior in relationship to alcohol abuse and microbial changes, especially in the case of addictive behavior such as alcohol-seeking behavior. Nevertheless, we are still convinced that more studies generated directly in humans and well-designed clinical trials need to be carried out to elucidate the complex interactions between the gut, the immune system and the brain and to better reflect the combined effects of excessive alcohol consumption together with all other parameters affected by alcohol dependence. To date, how alcohol induces a leaky gut and intestinal dysbiosis in some, but not all alcoholics, despite similar amounts of alcohol consumed, remains largely unknown. One could hypothesize that initial difference in the microbial composition may exist before the development of alcoholism in some susceptible patients. Only longitudinal prospective studies could answer this question.

The modulation of gut microbiota—by the use of pro- or prebiotics—and its effect on gut barrier, inflammation and the different behavioral aspects of addiction should be tested in rigorously conducted, placebo-controlled clinical trial. As diet is a major factor influencing the gut microbiota composition, dietary assessments should be carefully reported when performing clinical studies. Potential confounding factors such as obesity, diabetes and inflammatory bowel disease should be excluded. Finally, future studies should be targeted to better analyze the brain inflammatory response in relation with functional changes in the various brain circuits implicated in addiction in humans. Due to obvious ethical reasons, the effect of chronic alcohol consumption on brain inflammation has been exclusively studied in animal models. Therefore, the use of newly developed, inflammatory-specific tracers for functional magnetic resonance imaging or PET scan^159^ will definitely help to explore the mechanisms underlying peripheral immune-to-brain communication in humans.

## Figures and Tables

**Table 1:** Current pharmacological treatments of alcohol use disorders

*Pharmacotherapies*	*Mechanisms of action*	*Authorities approval*
Naltrexone (Nalorex, Depade, ReVia Vivitrol)	•μ-Opioid receptor antagonist •Blocks β-endorphin release induced by alcohol	FDA approved
Nalmefene (Selincro)	•μ and δ-pioid receptor antagonist •κ-Opioid receptor partial agonist	EMA approved
Acamprosate (Campral, Aotal)	Still under investigation •Acts on GABA and glutamate neurotransmitter systems	FDA approved
Baclofen (Lioresal)	•GABA_B_ receptor agonist	Temporary recommendation issued by the French drug agency ANSM
Disulfiram (Antabuse)	Aversive agent •Aldehyde dehydrogenase inhibitor (blocks the metabolism of alcohol’s primary metabolite acetaldehyde)	FDA approved

Abbreviations: ANSM, Agence nationale de sécurité du medicament; EMA, European Medicines Agency; FDA, Food and Drug Administration; GABA: γ-aminobutyric acid.

**Table 2:** Changes in intestinal microbiota associated with alcohol dependence in humans

*Subjects and stage of liver disease*	*Samples*	*Method to assess gut microbiota*	*Main bacterial changes in alcoholic subjects*				*Remarks*	*Ref*
			*Gram −*		*Gram +*			
			*Increase*	*Decrease*	*Increase*	*Decrease*		
Alcoholic subjects with mild liver injury/alcoholic hepatitis (*n*= 66, 100% males)	Feces	Quantitative culturing of stool samples				*Bifidobacterium* *Lactobacillus* *Enterococcus* vs healthy controls	1 week of abstinence did not restore the gut microbiota	^81^
Group ALD: subjects with mild liver disease (*n*=19, 63% males; 74% with Child–Pugh class of A). This group included active drinkers (*n*=8) and sober alcoholics (*n*=11) Group ALC: subjects without liver disease (*n*=29, 79% males). This group included active drinkers (*n*=14) and sober alcoholics (*n*=15)	Mucosal biopsy of the sigmoid colon	LH-PCR and MTPS of 16 S rRNA gene 27% of patients were identified as dysbiotic	Dysbiotic vs non-dysbiotic: *γ-Proteobacteria* *Sphingobacteria* *Verrucomicrobiaceae*	*Bacteroidaceae* vs healthy controls Dysbiotic vs non-dysbiotic: *Bacteroidetes*	Dysbiotic vs non-dysbiotic: *Bacilli*	Dysbiotic vs non-dysbiotic: *Clostridia*	No difference in serum LPS between ALD and ALC Only a subgroup of alcoholic subjects (with and without liver disease, active and sober) has altered colonic microbiota composition compared with healthy controls The dysbiotic cases had a higher frequency of diabetes (45% vs 3% in the dysbiotic vs nondysbiotics)	^26^
Alcoholic subjects with the absence of or minimal liver fibrosis (F0–F1), split into two subgroups: Low intestinal permeability (*n*=7, 71% males), non-dysbiotic High intestinal permeability (*n*=6, 50% males), dysbiotic	Feces	454 pyrosequencing of 16 S rRNA gene and quantitative PCR			Dysbiotic vs non-dysbiotic: *Lachnospiraceae* (*Dorea*) *Blautia* *Megasphaera*	Dysbiotic vs non-dysbiotic: *Bifidobacterium* *Clostridia* *Ruminococcaceae* (*Faecalibacterium prausnitzii*, *Ruminococcus*, *Subdoligranulum*, *Oscillibacter*, *Anaerofilum*)	Only alcoholic subjects with high intestinal permeability were identified as dysbiotic 3 weeks of abstinence did not restore the gut microbiota	^27^

Abbreviations: LH-PCR, length heterogeneity PCR fingerprinting; LPS, lipopolysaccharide; MTPS: multitag pyrosequencing.
